# Case report: Compound heterozygosity in *PKLR* gene with a large exon deletion and a novel rare p.Gly536Asp variant as a cause of severe pyruvate kinase deficiency

**DOI:** 10.3389/fped.2022.1022980

**Published:** 2022-12-01

**Authors:** Minsun Kim, Seung Yeob Lee, Namsu Kim, Jaehyeon Lee, Dal Sik Kim, Joonhong Park, Yong Gon Cho

**Affiliations:** ^1^ Department of Pediatrics, Jeonbuk National University Medical School and Hospital, Jeonju, South Korea; ^2^ Department of Laboratory Medicine, Jeonbuk National University Medical School and Hospital, Jeonju, South Korea; ^3^ Research Institute of Clinical Medicine of Jeonbuk National University, Biomedical Research Institute of Jeonbuk National University Hospital, Jeonju, South Korea

**Keywords:** pyruvate kinase deficiency, PK enzyme activity, *PKLR* exon deletion, p.Gly536Asp, next-generation sequencing, copy number variation, multiplex ligation-dependent probe amplification

## Abstract

Red cell pyruvate kinase (PK) deficiency is the most common cause of hereditary nonspherocytic hemolytic anemia and the most frequent enzyme abnormality of the glycolytic pathway. To the best of our knowledge, this is the first Korean PK deficiency study that analyzes copy number variation (CNV) using next-generation sequencing (NGS). A 7-year-old girl with jaundice was admitted for evaluation of a persistent hemolytic anemia. The proband appeared chronically ill, showing a yellowish skin color, icteric sclera, hepatomegaly, and splenomegaly on physical examination. Sequence variants and CNV generated from NGS data were estimated to determine if there was a potential genetic cause. As a result, compound heterozygosity in the *PKLR* gene for a large exon deletion between exon 3 and exon 9 accompanied with a novel rare p.Gly536Asp variant located on exon 10 was identified as a cause of severe PK deficiency in the proband. The PK activity of the proband had been measured at the time of day 1, 21, and 28 after receiving transfusion to indirectly assume the effect of the transfused blood, and the results were 100.9%, 73.0%, and 48.5%, compared with average of normal controls, respectively. Our report emphasizes the need to perform complete CNV analysis of NGS data and gene dosage assays such as multiplex ligation-dependent probe amplification to evaluate large deletions or duplications/insertions of the *PKLR* gene in patients with suspected PK deficiency.

## Introduction

Red cell pyruvate kinase (PK) deficiency (OMIM # 266200) is the most common cause of hereditary nonspherocytic hemolytic anemia. It is also the most frequent enzyme abnormality of the glycolytic pathway (1). PK plays a central role in erythrocyte metabolism. As a glycolytic enzyme, PK is encoded by the *PKLR* gene. It catalyzes the transphosphorylation from phosphoenolpyruvate to adenosine diphosphate (ADP), yielding pyruvate and adenosine triphosphate (ATP). It is the last step of the glycolytic pathway and is essentially irreversible. Therefore, PK deficiency can lead to ATP depletion, which ultimately affects cell viability (1). The prevalence of PK deficiency in epidemiological studies of hereditary hemolytic anemia (HHA) in a Korean pediatric population from 1997 to 2016 was calculated to be 1.85% (13/702) (2, 3). Clinical manifestations of PK deficiency are extremely variable, ranging from severe forms associated with intrauterine death, hydrops fetalis, or neonatal jaundice requiring exchange transfusions to fully compensated hemolytic anemia (4). Endocrinopathies, leg ulcers, bone disease, pulmonary hypertension, gallstones, and iron overload are common complications as well (5). Treatment options include chelation therapy, splenectomy, and red blood cell (RBC) transfusion. Hematopoietic stem cell transplantation has been performed in very few cases (6). It has been demonstrated that oral administration of mitapivat, an investigational drug and a small molecule allosteric activator of pyruvate kinase in red cells, can improve hemoglobin level and hemolysis markers in patients with at least one *PKLR* missense variant (7).

Clinical PK deficiency is transmitted as an autosomal recessive trait, which can segregate either in compound heterozygous or in homozygous modality. Two different *PKM2* and *PKLR* genes are present in mammals: (1) pyruvate kinase muscle (*PK-M*) and (2) pyruvate kinase liver and RBCs (PK-LR). Only the second kind encodes for isoenzymes normally expressed in RBCs (R-type) and the liver (L-type) (8). The number of known pathogenic *PKLR* variants is continuously increasing. In sequence databases such as the Human Genome Mutation Database (HGMD, https://www.hgmd.cf.ac.uk/) and Leiden Open Variation Database (LOVD, https://databases.lovd.nl/shared/genes/PKLR), more than 300 various *PKLR* variants have been reported, and the study of the diagnostic impact of genotypes and the extensive molecular heterogeneity of PKD have been examined (9). However, mutations in consensus splice sites and deletions in the 5′UTR involving the *GATA1* binding site or of whole exon 11 have also been found. Among these mutations, single nucleotide substitutions are the most common (up to 72%), followed by indels (up to 13%). Several mutations in introns and promoter/enhancer regions have also been found (4, 8, 9).

To date, several Korean cases of PK deficiency caused by *PKLR* mutations have been reported (6, 10, 11). To the best of our knowledge, this is the first to analyze copy number variation (CNV) in a Korean patient with PK deficiency using next-generation sequencing (NGS). *PKLR* exon deletion accompanied by a novel missense *PKLR* variant in a patient with severe phenotype of PK deficiency was identified.

## Case presentation

**Figure 1:**
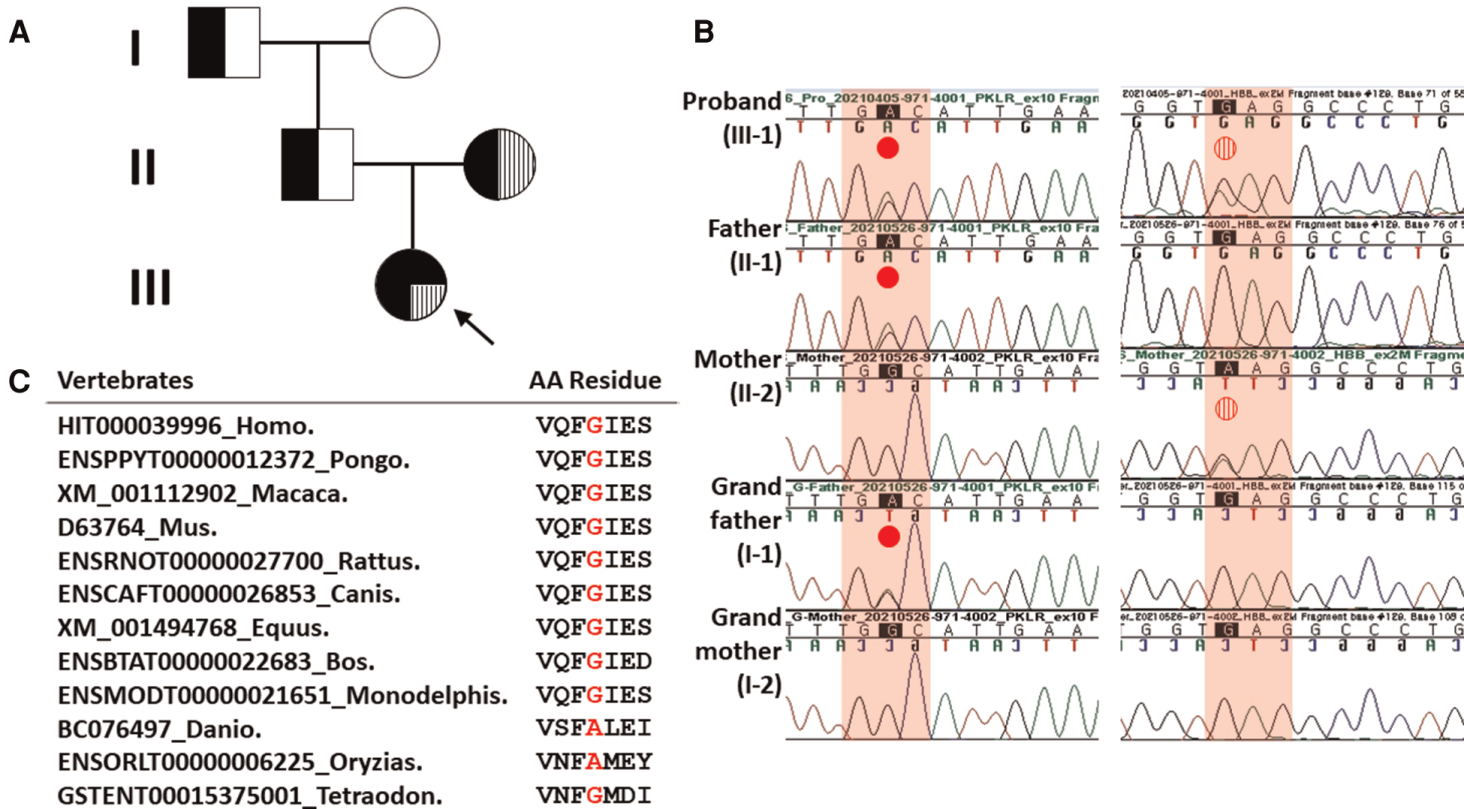
Pedigree analysis and Sanger sequencing results of heterozygous missense *HBB* and *PKLR* mutations in the proband and her family members. (**A**) Family pedigree showing that heterozygous missense *HBB* mutation and *PKLR* variant in the proband were inherited *in trans*, respectively. Solid black dot, *PKLR* variant; filled vertical line, missense *HBB* mutation. (**B**) In the left column, a missense *PKLR* variant (NM_000298.6: c.1607G>A, p.Gly536Asp) was inherited with a paternal origin (indicated by solid red dot). In the right column, a missense mutation (NM_000518.5: c.79G>A, p.Glu27Lys; rs33950507) of the *HBB* was inherited with a maternal origin (indicated by vertical striped red dot). (**C**) Sequence alignment of the PKLR protein in multiple vertebrate species. Protein sequence of the Gly536 residue is highly conserved between Human and Tetraodon except Danio and Oryzias analyzed by Evola (http://www.h-invitational.jp/evola/). It is highlighted in the red letter.

A 7-year-old girl (III-1 in Figure 1A) with jaundice, a firstborn female of healthy parents (Korean male and Vietnamese female) at 39 weeks of gestation, visited the Division of Endocrinology, Department of Pediatrics, Jeonbuk National University Hospital (Jeonju, South Korea), for evaluation of a persistent hemolytic anemia. The proband appeared chronically ill with yellowish skin color, icteric sclera, hepatomegaly, and splenomegaly on physical examination. The rest of the examination was unremarkable. She was born at 38 weeks by vaginal delivery, with a birth weight of 2,300 g and unremarkable perinatal history. She was admitted to the neonatal intensive care unit after birth due to a lethargic condition. She was diagnosed with fetomaternal transfusion with suspected HHA. She was transfused with packed red cells. As time went on, she became pale and bright yellowish skin discoloration progressively. There was no family history of hemolytic disease. On the date of birth, her initial investigations showed a hemoglobin level of 5.3 g/dl, mean cell volume of 122.7 fl, total leukocyte count of 29,780/mm^3^, platelets of 159,000/mm^3^, and reticulocyte percentage of 60.18%. Direct Coombs test result was positive. A peripheral blood smear test showed marked leucoerythroblastic reaction and atypical lymphoid cells (1%–2%). Total bilirubin level was 12.51 mg/dl (normal range, 0.2–1.2 mg/dl), of which direct bilirubin level was 0.94 mg/dl (normal range, 0–0.4 mg/dl). Her aspartate aminotransferase and alanine aminotransferase levels were 107 IU/l (normal range, 24–95 IU/l) and 22 IU/l (normal range, 6–40 IU/l), respectively. Based on signs and symptoms including mild to severe anemia, paleness, fatigue, yellow discoloration of skin (jaundice), and bone problems, thalassemia was suspected initially in the proband. Sanger sequencing for *HBB* and *HBA1/2* was performed at the Green Cross Genome (Yongin, South Korea). As a result, heterozygous missense mutation (NM_000518.5: c.79G>A, p.Glu27Lys; rs33950507) of the *HBB* was detected in both the proband and her mother (II-2 in Figure 1A). Although her mother had the same heterozygous *HBB* gene mutation, she showed no hemolytic symptoms. Thus, the proband and her mother were diagnosed with beta thalassemia trait. This trait usually has no clinical manifestation. The proband has been receiving RBC transfusions 1–2 times a month since birth until recently. On the other hand, the reticulocyte count of the proband was 1.64% at the time of the PK activity measurement. The red cell count and hematocrit were 1.88 × 10^6^/μl and 16.5%, respectively; thus, the absolute reticulocyte count was 30,832/μl and the approximate corrected reticulocyte count percent was 0.6%.

Because the proband's parents and other family members presented no clinical symptoms associated with HHA, genetic counseling and segregation analysis were additionally performed to identify other genetic causes besides the *HBB* gene.

## Materials and methods

### Singleton next-generation sequencing

This study was reviewed and approved by the Institutional Review Board (IRB) of Jeonbuk National University Hospital (Approval number: 2021-09-039; Date of approval: September 16, 2021). Written informed consent was collected from the parents on behalf of their children for clinical and molecular analyses and for the publication of any potentially identifiable images or data included in this study. To determine the potential genetic cause of the suspected HHA in our proband, her gemonic DNA was analyzed by NGS with the Celemics G-Mendeliome Clinical Exome Sequencing Panel (Celemics, Seoul, South Korea). This panel targets exonic regions that harbor disease-causing variants in up to 5,870 genes associated with known clinical phenotypes according to the Human Gene Mutation Database (HGMD, http://www.hgmd.cf.ac.uk/ac/index.php), GeneTests (www.genetests.org), and Online Mendelian Inheritance in Man (OMIM, www.omim.org). Particularly, *ABCB7, AK1, ALAS2, ANK1, EPB41, EPB42, GLRX5, G6PD, GCLC, GPI, GPX1, GSR, GSS, HK1, KCNN4, NT5C3A, PGK1, PIEZO1, PKLR, PUS1, RHAG, SCL25A38, SLC19A2, SLC4A1, SPTA1, SPTB, TMPRSS6, TPI1, UGT1A1*, and *YARS2* genes responsible for HHA were included. Paired-end sequencing on a NextSeq500 instrument (Illumina, San Diego, CA, United States) with a flow cell high output, 300 cycles PE (150 bp × 2) at the Green Cross Genome was conducted to detect the variant given the suspicion of HHA. Base calling, alignment, variant calling, annotation, and quality control reporting were performed using the Genome Analysis Tool Kit best practice pipeline workflow for germline short variant discovery (https://gatk.broadinstitute.org/hc/en-us). Interpretation of sequence variants was manually reviewed by medical laboratory geneticists according to standards and guidelines from the Joint Consensus Recommendation of the American College of Medical Genetics and Genomics and the Association for Molecular Pathology (12). Because deletions and duplications of causative genes are associated with HHA (3), CNV analysis from raw sequencing data was performed by VisCap (13), mainly used, and ExomeDepth (14) used as an auxiliary. Briefly, the sample-specific fractional coverage of each region is divided by the median for that region across the entire batch, where a batch refers to a set of 30 samples provides a representative median coverage across all target regions. The cutoffs to identify a loss or a gain depending on the distribution of log_2_ ratios for each sample are defined as the minimum log_2_ ratio for losses of −0.55 and maximum log_2_ ratio for gains of 0.40. The boxplot interquartile range multiplier was set at 3 (13).

### Multiplex ligation-dependent probe amplification

Multiplex ligation-dependent probe amplification (MLPA) was performed to confirm the large exon deletion of the *PKLR* identified by NGS with CNV analysis using a SALSA® MLPA® Probemix P203-B2 PKLR (MRC-Holland, Amsterdam, The Netherlands) according to the manufacturer's instruction. Capillary electrophoresis and fragment analysis were conducted on a 3730XL Genetic Analyzer (Applied Biosystems, Carlsbad, CA, United States). Comparative CNV data were analyzed using Coffalyser.Net software ver. 210604.1451 (MRC-Holland) according to the manufacturer's protocol. Resulting peak intensities were normalized to those of sex-matched normal DNA and manufacturer control probes as a reference. Assuming that the probe normally targets two copies, a probe to peak ratio of 0.8–1.2 was defined as a normal copy number (wild-type). A probe to peak ratio of 0.4–0.65 represented a heterozygous deletion (loss of one copy number). On the other hand, probe results indicated a heterozygous duplication when an increase of more than two standard deviations was found and the upper arbitrary border of 1.3 was crossed.

### Pyruvate kinase enzyme activity assay

PK enzyme activity was tested using a Pyruvate Kinase Activity Assay Kit (Sigma-Aldrich, St. Louis, MO, United States) in spectrophotometric detection at 570 nm according to the instructions of the manufacturer (15). The test kit provides results as mU/ml (nmol/min/ml) where one unit of PK is defined as the amount of enzyme transferring a phosphate from phosphoenolpyruvate to ADP to generate 1.0 μmol of pyruvate per minute at 25 °C. To reduce storage time of the sample, EDTA-treated blood was centrifuged immediately after sampling. After removing buffy-coat and plasma from the blood sample, the pallet of RBCs was mixed with the distilled water to test the PK activity of erythrocyte lysate. Fourteen, twelve, and two healthy individuals were tested in parallel as normal controls at the time of 1, 21, and 28 days after the proband received the transfusion, respectively. The PK activity of the proband's father was simultaneously measured along with the proband, on days 1 and 21 after the proband received the transfusion. All samples and standard solutions were tested in duplicate. The PK activity results were interpreted as a percentage of the average PK activity of normal controls with a 95% confidence interval (CI) rather than the results in mU/ml, because the Pyruvate Kinase Activity Assay Kit used to measure PK activity on different days had different lot numbers, which might provide different values.

## Results

A yield on target of 5,181,268,698 reads was generated from the proband's sample by estimating the sequence quality along all sequences in the NGS. The mean read depth (×) was 261. The percentage of bases above read depth of 30× was 97%. One variant with uncertain significance (VUS) of the *PKLR* (left column in Figure 1B) and one known pathogenic *HBB* mutation detected previously by Sanger sequencing (right column in Figure 1B) were identified. Sanger sequencing was conducted to discriminate between inherited and noninherited sporadic hemolytic anemia as a genetic origin. As a result, this VUS of the *PKLR* was identified in the proband, her father (II-1 in Figure 1A), and her grandfather (I-1 in Figure 1A). This missense *PKLR* variant located on exon 10 (NM_000298.6: c.1607G>A, p.Gly536Asp; GenBank accession number: OP404085) was not reported in 1,722 Korean normal individuals (KRGDB, http://coda.nih.go.kr/coda/KRGDB/index.jsp) or the Genome Aggregation Database (gnomAD, https://gnomad.broadinstitute.org). This novel missense variant occurring at the same position as another missense change (p.Gly536Val; rs1572051895) reported as a VUS previously was considered a moderate evidence (PM5). However, it could not be assumed to be pathogenic. This is especially true if the novel change is more conservative than the established pathogenic missense variant. Different amino acid changes could also lead to a different phenotype (12). Multiple lines of computational evidence support a deleterious effect on this VUS of the *PKLR* classified as PP3 by ACMG criteria for classifying pathogenic variants (12). It was predicted to be disease causing or damaging with Condel (damaging with a score of 0.664 when a cutoff of 0.522 applied, http://bbglab.irbbarcelona.org › fannsdb), PolyPhen-2 (possibly damaging with a score of 0.975, http://genetics.bwh.harvard.edu/pph2/), Fathmm v2.3 (damaging with a score of 0.975, http://fathmm.biocompute.org.uk/), CADD (harmful with a PHRED score of 27.3 when a cutoff of 20 applied, https://cadd.gs.washington.edu/) (16), MutationTester (disease causing with a score of 94, https://www.mutationtaster.org), and SIFT (not tolerated with a score of 1, https://sift.bii.a-star.edu.sg/). Furthermore, it was predicted to be highly conserved with values of 3.568 and 1 by PhyloP and PhastCons, respectively. Protein sequence of the Gly536 residue is highly conserved between Human and Tetraodon except Danio and Oryzias analyzed by Evola (http://www.h-invitational.jp/evola/) (Figure 1C). Variant allele frequency was assessed using public sequence databases from genome aggregation database (gnomAD, https://gnomad.broadinstitute.org/) and Korean reference genome database, the large-scale variant database of 1,722 Koreans based on whole genome sequencing (KRGDB, https://coda.nih.go.kr/coda/KRGDB/). This novel p.Gly536Asp variant of the *PKLR* has previously has not been observed in the general population (gnomAD) and the Korean ethnic population (KRGDB).

**Figure 2:**
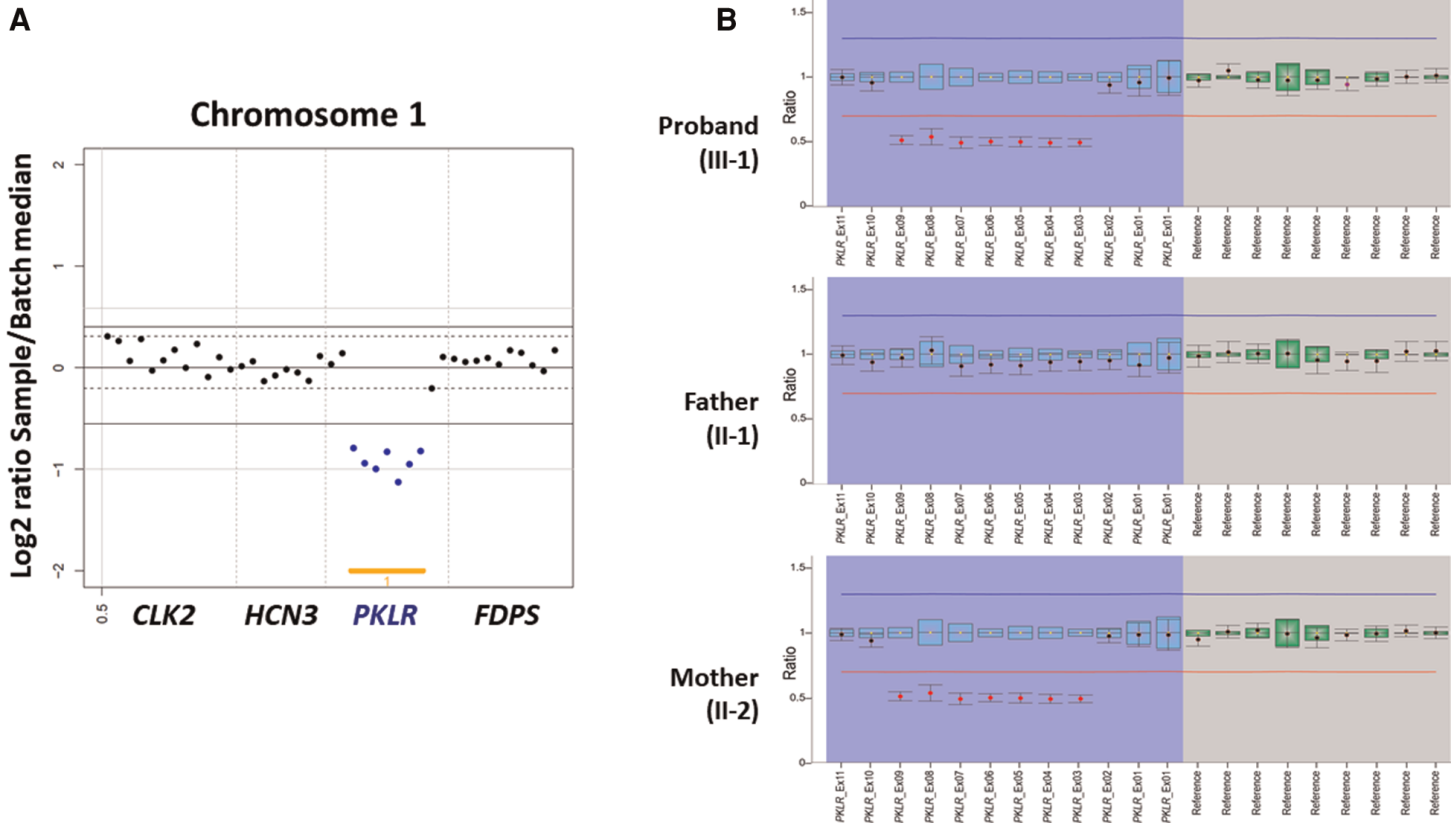
Heterozygous *PKLR* deletion identified by copy number variation (CNV) analysis using next-generation sequencing and multiplex ligation-dependent probe amplification (MLPA) in a proband with pyruvate kinase deficiency. (**A**) VisCap, a copy number detection and visualization tool, illustrated a heterozygous 2.6 Kb deletion of chromosome 1q22 (chr1:155,262,957-155,265,561), including a region between exon 3 and exon 9 of the *PKLR* with a Z ratio of −0.5 to −1 in the proband (indicated by solid blue dots under than lower normal limit of −0.5). (**B**) MLPA demonstrating the same heterozygous deletion identified previously by CNV analysis with NGS data in the proband and her mother (indicated by solid red dots under than lower normal limit of 0.65).

On the other hand, both CNV analysis tools detected a heterozygous 2.6 Kb deletion of chromosome 1q22 (chr1:155,262,957–155,265,561), including a region between exon 3 and exon 9 of the *PKLR* with a Z ratio of −0.5 to −1 in the proband (Figure 2A). MLPA was additionally performed to confirm this *PKLR* exon deletion in the proband and her family members. As a result, the same heterozygous deletion identified previously by CNV analysis based on NGS data was confirmed in the proband and her mother (Figure 2B). Based on genetic testing results of family studies, the missense *PKLR* variant located on exon 10 and the large deletion between exon 3 and exon 9 of the *PKLR* were *in trans* nature.

To evaluate the functional effect of the *PKLR* mutation, PK activities of the erythrocytes of the proband harboring compound heterozygous mutation and her father harboring sole missense mutation were tested. Since the proband has severe anemia and is receiving regular transfusions, the PK activity testing result of the proband's erythrocytes could be affected by transfused erythrocytes. Thus, the PK activity of the proband had been measured at the time of day 1, 21, and 28 after receiving transfusion to indirectly assume the effect of the transfused blood, and the results were 100.9% (90.4%–114.3% of 95% CI; 220.9 mU/ml of the proband vs. average 218.9 mU/ml of 14 normal controls), 73.0% (64.5%–84.1% of 95% CI; 214.5 mU/ml of the proband vs. average 293.7 mU/ml of 12 normal controls), and 48.5% (44.9%–52.8% of 95% CI; 272.1 mU/ml of the proband vs. average 560.7 mU/ml of two normal controls) of normal controls, respectively. The proband's father harboring sole missense mutation but no anemia showed 44.5% (39.8%–50.4% of 95% CI; 97.4 mU/ml of the proband's father vs. average 218.9 mU/ml of 14 normal controls) and 35.4% (31.3%–40.8% of 95% CI; 104.0 mU/ml of the proband's father vs. average 293.7 mU/ml of 12 normal controls) PK activity compared with normal controls in independent testing days.

Taken together, our proband with HHA was diagnosed properly with PKD caused by compound heterozygous *PKLR* mutation rather than a beta thalassemia trait caused by heterozygous missense *HBB* mutation based on genetic study results, decreased PK activity of erythrocytes, and severe hemolytic symptoms.

## Discussion

*PKLR* gene codes for both PK-L and PK-R isoenzymes using two alternative promoters. Exons 1 and 2 of the *PKLR* gene are specifically transcribed to PK-R and PK-L mRNAs, respectively, while nine exons from exon 3 to exon 11 are shared by the two isoforms (17, 18). Although abnormalities in the *PKLR* gene may result in alterations of both erythrocytes and liver enzymes, clinical symptoms are confined to RBCs. PK-deficient patients usually can tolerate anemia well. Thus, the decision to transfuse or treat a patient is based on how the patient feels rather than on an arbitrary hemoglobin threshold (19). This is in part justified by increased 2,3-diphosphoglycerate level typically found in these patients. As an important regulator of oxygen affinity of hemoglobin, 2,3-diphosphoglycerate may enhance oxygen delivery (20).

Genetic analysis of the *PKLR* gene is necessary to confirm the diagnosis and overcome limitations of enzymatic testing, which may show false-negative results in the case of an increased number of reticulocytes, recent transfusion, or false-positive results in the case of heterozygous carriers. Therefore, recently consensus recommendations propose that enzymatic testing and genetic analysis are complementary techniques for diagnosing PK deficiency (15). However, there are many cases suspected to have PK deficiency on clinical grounds, with only a single mutant allele identified or carried large insertions/deletions, deep/junctional intronic, or promoter/enhancer variants (6, 9, 21), which require more comprehensive molecular analysis. NGS is increasingly used for genetic diagnosis of patients presenting with HHA including RBC enzymopathy because it allows simultaneous interrogation of multiple complement and coagulation pathway genes known to be associated with HHA (22–25). Although gene panel or exome sequencing has been widely used by medical laboratory to detect small coding variants, it might miss large insertions/deletions, deep/junctional intronic, or promoter/enhancer regions. It is very difficult to ascertain the existence of large insertion or deletion responsible for HHA and may require specific testing such as complex minigene construct approach or loss of heterozygosity by analyzing an allele-specific cDNA (9). Current best practices for CNV detection often require the use of MLPA for gene-sized CNVs or chromosomal microarrays to detect large CNVs > 50 Kb. In addition to sequencing a clinically enhanced exome to enable targeted disease-specific variant analysis, application of CNV detection algorithms using various tools in routine targeted NGS diagnostic settings can facilitate immediate improvement in clinical care for individuals with heterogeneous genetic diseases (13, 26, 27). Thus, NGS panel data can be used as a CNV screening step in a genetic diagnostic setting. This screening step has the potential to improve genetic diagnosis of PK deficiency.

A few large *PKLR* deletions have been reported (4, 28); in East Asia, a Vietnamese family with a large deletion extending from exon 4 to exon 10 (29) and a Chinese family with a large deletion (c.283+1914_c.1434del5006) (30) were identified by long-range polymerase chain reaction. The other exon 4 to exon 11 deletion of *PKLR* identified in the same Chinese family is the largest deletion that predictably results in no functional enzyme production. Similar to this, in our case, segregation analysis from family members suggests that a large *PKLR* deletion was transmitted by the proband's mother to the proband. Thus, it occurred independently for several times in East Asia or that the founder deletion occurred in Chinese, which was subsequently spread by Chinese migrants (30). On the other hand, PK M2, L, and R isoenzymes display sigmoidal reaction kinetics with respect to phosphoenolpyruvate. They are allosterically activated by fructose 1,6-bisphosphate (FBP) (31). Biochemical characterization and crystal structure analysis of recombinant erythrocyte PK of eight mutants have shown that molecular properties of mutant enzymes are correlated with clinical symptoms (31). Among eight mutants, p.Arg532Trp that is close to p.Gly536Val, another responsible variant for PK deficiency in our case is located on the allosteric site. It is fully unresponsive to FBP, highlighting the pivotal role of Arg532 in activator binding. The lack of allosteric properties is associated with a decreased thermostability, possibly reflecting the energetically unfavorable exposure on the protein surface of the hydrophobic Trp residue (31). Clinical studies have investigated genotype–phenotype correlation and found that patients with severe symptoms more frequently harbor missense pathogenic variants or nonsense mutations affecting the stability or active site of the PK protein (32–34). An international multicenter study on genotype–phenotype correlation has revealed that patients with non-missense (NM)/NM mutations have a more severe phenotype with a higher rate of iron overload, a higher number of transfusions throughout their lifetime, a higher rate of splenectomy, and lower hemoglobin levels after splenectomy than patients with missense/NM or missense/missense *PKLR* mutations (28). Although our case is classified as a missense/NM, the combination of a missense mutation close to the allosteric site and a large exon deletion between exon 3 and exon 9 might lead to severe clinical manifestations.

Although a systematic study of PK activity in PK deficiency patients has not been reported yet, one study showed that the mean PK activity of erythrocytes in PK deficiency cases was 38.5% of that in the controls (15). Another study showed a median 1.1 enzyme unit (eu)/g hemoglobin Hb (ranging from 0.2 to 4.6) PK activity in 41 PK deficiency patients when reference range was 3.2 to 6.5 eu/g Hb (35). Regarding the reference interval, there is no absolute range because of diverse protocols including assay temperature and substrates. A reference interval at 4.6–11.2 U/g Hb was established in one study, whereas a reference interval at 11.1–15.59 IU/g Hb was set in another study (36, 37). Similarly, the PK activities of normal controls were also different along with different test kits and measurement days in the current study. The average values of normal controls ranged from 218.9 to 560.7 mU/ml according to different lots of the test kits. When converting the PK activity unit of mU/ml to U/g Hb at the time of 4 weeks after transfusion, the calculated value was 0.86 U/g Hb for the proband and an average of 1.71 U/g Hb (1.59–1.83 U/g Hb of 95% CI) for normal controls. Considering the lifespan of erythrocytes at 120 days, remnant donor erythrocytes with normal PK activity might have falsely elevated the patient's PK activity (15). Although it was impossible to measure the PK activity of the proband's pure erythrocytes due to regular transfusion, it is reasonable to infer that the actual activity would be much lower than 50% of normal controls when considering the decreased PK activity approximately from 100% to 50% as the increased elapsed period after transfusion from day 1 to 28. Moreover, the low PK activity (approximately 40% of normal controls) of the proband's father who has only sole missense mutation without symptom also supports the very low PK activity of the proband harboring compound heterozygous mutation with severe hemolysis and anemia. Therefore, although these values could not be directly compared with previous reports due to different test protocols, the current case showed a reduced enzyme activity compatible with a PK deficiency patient.

In conclusion, we genetically diagnosed compound heterozygosity in the *PKLR* gene for a large exon deletion between exon 3 and exon 9 accompanied with a novel rare p.Gly536Asp variant located on exon 10 as a cause of severe PK deficiency in a Korean girl. Our report emphasizes the need to perform complete CNV analysis of NGS data and gene dosage assays such as MLPA to evaluate large deletions or duplications/insertions of the *PKLR* gene in patients with suspected PK deficiency.

## Data Availability

The original contributions presented in the study are included in the article/**Supplementary Material**, further inquiries can be directed to the corresponding authors.
