# Supplementary material for: Case report: Compound heterozygosity in PKLR gene with a large exon deletion and a novel rare p.Gly536Asp variant as a cause of severe pyruvate kinase deficiency
Source: Front Pediatr. 2022 Dec 1;10:1022980. doi: 10.3389/fped.2022.1022980 (PMC9752143; doi:10.3389/fped.2022.1022980)
Supplement: Supplementary file 1 [file Datasheet1.docx]

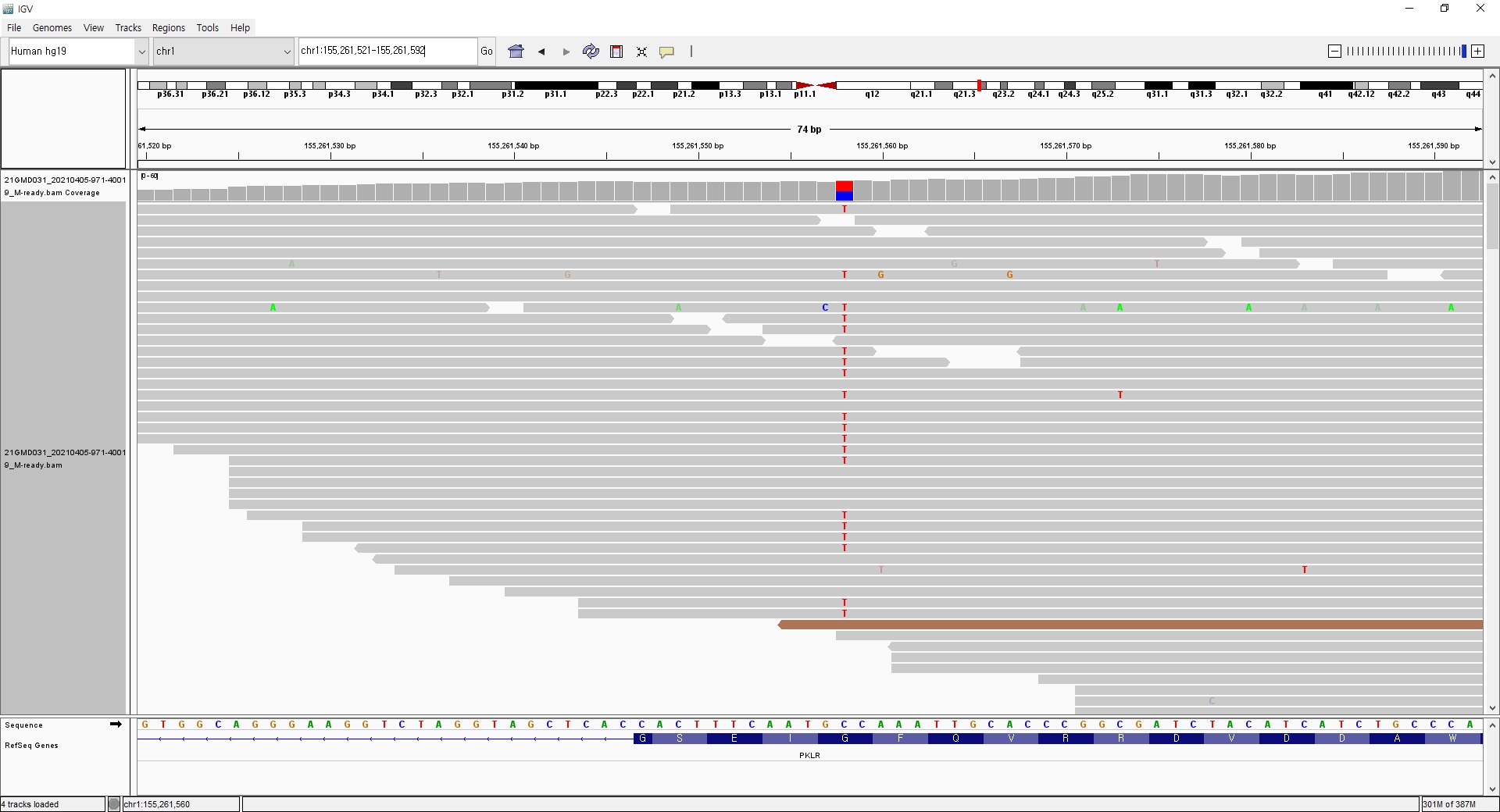


**Supplementary Figure S1.** Identification of a missense *PKLR* variant (NM_000298.6: c.1607G>A, p.Gly536Asp) by next-generation sequencing (NGS) in the proband. Visualization of *PKLR* sequencing reads containing p.Gly536Asp with the Integrative Genomics Viewer (IGV).


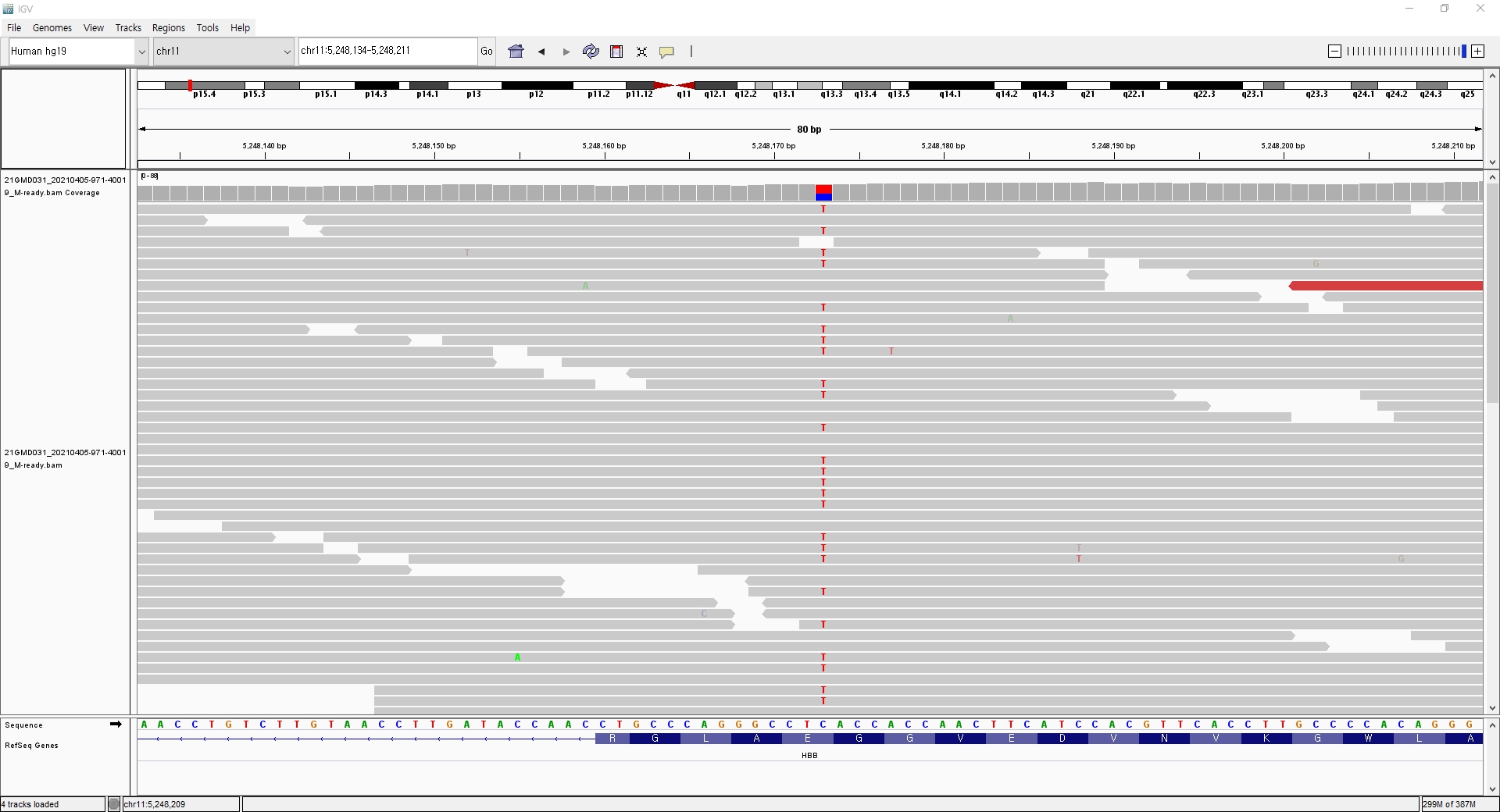


**Supplementary Figure S2.** Identification of a missense *HBB* variant (NM_000518.5: c.79G>A, p.Glu27Lys) by next-generation sequencing (NGS) in the proband. Visualization of *HBB* sequencing reads containing p.Glu27Lys with the Integrative Genomics Viewer (IGV).
